# Can Stray Cats Be Reservoirs of Antimicrobial Resistance?

**DOI:** 10.3390/vetsci9110631

**Published:** 2022-11-12

**Authors:** Valeria Gargano, Delia Gambino, Tiziana Orefice, Roberta Cirincione, Germano Castelli, Federica Bruno, Paolo Interrante, Mariangela Pizzo, Eva Spada, Daniela Proverbio, Domenico Vicari, Marília Salgado-Caxito, Julio A. Benavides, Giovanni Cassata

**Affiliations:** 1 Istituto Zooprofilattico Sperimentale della Sicilia “A. Mirri”, 90129 Palermo, Italy; 2 Department of Veterinary Medicine and Animal Sciences (DIVAS), University of Milan, 26900 Lodi, Italy; 3 Escuela de Medicina Veterinaria, Facultad de Agronomía e Ingeniería Forestal, Facultad de Ciencias Biológicas y Facultad de Medicina, Pontificia Universidad Católica de Chile, Santiago 7500000, Chile; 4 Centro Para la Investigación de la Sustentabilidad y Doctorado en Medicina de la Conservación, Facultad Ciencias de la Vida, Universidad Andres Bello, República 440, Santiago 8320000, Chile; 5 MIVEGEC, IRD, CNRS, Université de Montpellier, 34394 Montpellier, France

**Keywords:** domestic animals, resistance genes, *E. coli*, antibiotic resistance, One Health, Italy, extended-spectrum beta-lactamases

## Abstract

**Simple Summary:**

The spread of antimicrobial resistance is the main cause of failure in the treatment of infectious diseases. Antimicrobial resistance can transmit from host to host, even with low numbers of bacteria, and the exchange of antibiotic resistance genes can occur between bacteria in animals and humans. The role of animals as vectors and reservoirs of antimicrobial resistance is a growing global problem. Several studies have demonstrated the presence of antimicrobial resistance in companion and food-producing animals, but few in strays. In urban environments, stray cats are on the borderline between pets and strays and may play a role in the spread of antimicrobial resistance. In order to investigate the possible role of these animals, we evaluated antibiotic resistance and the presence of resistance genes of *E. coli* isolated from feces of stray cats. *E. coli* were resistant to beta-lactams and tetracyclines, two important classes of antibiotics, and harboured genes encoding these resistances. Although further investigation is needed, the presence of resistant *E. coli* confirms the hypothesis that stray cats may be faecal carriers of antibiotic resistance and the need to consider these animals in antimicrobial resistance monitoring programmes.

**Abstract:**

The emergence and spread of antimicrobial resistance (AMR) is a global problem that requires a One Health approach. Despite several studies have reported the role of companion animals as reservoirs of AMR, limited information is available regarding the role of cats in the circulation of AMR. In this study, we evaluated the phenotypic and genotypic profile of 75 *Escherichia coli* isolated from rectal swabs and fecal samples of 75 stray cats (living in solitary or in a colony) sampled in Palermo (Sicily, Italy), to determine whether these animals may participate in the spread of AMR. Susceptibility to 8 antibiotics was tested using Minimum Inhibitory Concentration assays, while the presence of the common antibiotic resistance genes *bla*_TEM_, *bla*_CTX-M_, *tet*(A), and *tet*(B) was investigated by PCR. From the 75 *E. coli* isolates analyzed, 43% were resistant to at least one of the eight antibiotics tested, with 31% of the isolates resistant to ampicillin, 23% to cefotaxime, 21% to tetracycline, 20% to cefazolin, and 17% to amoxicillin/clavulanic acid. Most isolates harbored the *bla*_TEM_ gene (29%), followed by *bla*_CTX-M_ (23%), *tet*(A) (21%), and *tet*(B) (20%). Our results confirm the fecal carriage of antibiotic-resistant *E. coli* and clinically relevant resistance genes in stray cats. This study highlights the potential role of stray cats in the spread of AMR in urban environments, emphasising the need to better understand their role in AMR circulation when planning strategies to combat it.

## 1. Introduction

Although antibiotics have contributed to treat millions of animal and human infections, the development of antimicrobial resistance (AMR) mechanisms is now one of the main threats to public and animal health worldwide [1,2]. The emergence and spread of antimicrobial resistance is closely related to the uncontrolled use of antibiotics, not only in human and veterinary medicine, but also in the agricultural sector [2,3,4]. The widespread circulation and public health implications of AMR have prompted the scientific community, as well as national and international institutions, to monitor the presence and circulation of AMR among humans, animals (livestock, pets, and wildlife), and the environment following a One Health approach [5]. Consequently, since 2005, the World Health Organization (WHO) has compiled a list of Critically Important Antimicrobials (CIA) requiring surveillance to reduce the spread of AMR and preserve drugs important to human medicine which includes antibiotics that should not be used in veterinary medicine except under certain conditions [6].

In Europe, veterinary antimicrobial consumption is monitored by the ‘European Surveillance of Veterinary Antimicrobial Consumption’ project since 2010, which developed a harmonized approach for collecting and reporting data on the sale and use of antibiotics in animals [7]. However, the monitoring of AMR among animals is not systematic across Europe, or across all domestic animal species. For example, only 12 out of the 27 European countries have a national monitoring system of AMR in animals, which does not include Italy [8,9,10]. Furthermore, AMR monitoring programs have focused on food-producing animals, and no specific program exists for companion animals such as dogs and cats [8,9,10]. Thus, despite the excessive use of antimicrobial agents in these animals and their frequent contact with humans, the role of companion animals as potential reservoirs of AMR remains poorly understood [11,12].

*Escherichia coli* is a common commensal bacteria of the gastrointestinal tract of humans and warm-blooded animals, although it can also cause a variety of diseases. Because of their significant genetic diversity and great ability to accumulate antibiotic resistance genes (ARGs), especially through horizontal gene transfer, these bacteria act as a donor and a reservoir of ARGs [13]. The acquisition of genes encoding for beta-lactamases, particularly extended-spectrum beta-lactamases (ESBLs), that confer resistance to penicillins, aminopenicillins and third- and fourth-generation cephalosporins, is one of the most problematic resistance mechanisms in *E. coli* [14]. *E. coli* isolated from livestock animals are also commonly resistant to tetracycline [15]. Although the prevalence of ESBL-*E. coli* varies widely in different geogrephical regions, it is estimated to be 7% in dogs and 5% in cats worldwide, reflecting their potential role in the carriage and spread of these bacteria [16]. However, the number of studies focusing on cats is still very limited, which could result from lower interest in this species compared to dogs, but also logistical challenges such as difficulty in handling and sampling these animals or finding fecal simples, as cats burrow their feces [16,17,18].

Compared to owned animals, few studies have focused on the role of stray companion animals as disseminators of resistance to broad-spectrum antibiotics used in veterinary and human medicine, such as cephalosporins and tetracyclines [19]. In this study, we aimed to gain further insight into the role of stray cats in the dissemination of AMR on the island of Sicily. We evaluated the fecal carriage of antibiotic-resistant *E. coli* and ARGs to antibiotics still widely used in Sicily, including cephalosporins and tetracyclines, for the treatment of intracellular endemic pathogens (e.g., *Bartonella*, *Mycoplasma, Pasteurella multocida* and *Bordetella)* [20]. 

## 2. Materials and Methods

### 2.1. E. coli Isolation and Identification

From January to June of 2022, a rectal swab or a fresh faecal sample from 75 stray cats were collected in Palermo (Sicily, Italy). All cats were stray animals free-roaming either alone or in a group of cats (i.e., cat colony). Cats were captured for sterilization within the framework of the TNR (Capture-Sterilization-Release) programs under Italian law and screened for bacterial infectious diseases [21]. Thus, information on their health status and prior antibiotic treatments was unavailable.

Fresh faecal samples and rectal swabs were transported to the laboratory in AMIES transport medium (Biosigma S.p.A., Venice, Italy), and were processed within 48 h by seeding them on MacConkey agar plates (MC, Thermo Fisher Scientific, Waltham, MA, USA), a selective and differential medium for *Enterobacteriaceae*, incubated at 37 °C for 24 h. From each MC plate, one isolate with growth characteristics (pink colony; lactose fermenter) attributable to *E. coli* was randomly selected. Isolates suspected as *E. coli* were transplanted into brain-heart infusion agar incubated at 37 °C for 24 h, and confirmed as *E. coli* by biochemical tests including Gram stain, catalase test, oxidase test and indole production test, before confirming bacteria species identity by amplifying the 16S rRNA. Briefly, bacterial DNA was extracted from each isolate using the King Fisher automated extractor (Thermo Fisher Scientific Waltham, MA, USA) and the QIAamp One-For-All Nucleic Acid Kit according to the manufacturer’s instructions (QIAGEN Sciences, Germantown, PA, USA). For amplification of 16S rRNA, a reaction mix has been prepared using a TaqGold DNA polymerase (Thermo Fisher Scientific, Waltham, MA, USA) and primers U1 (5′-CCAGCAGCCGCGGTAATACG-3′) and U2 (5′-ATCGG(C/T)TACCTTGTTACGACTTC-3′) [11]. *E. coli* ATCC 25922 (American Type Culture Collection, Rockville, MD, USA) and nuclease-free water were used as positive and negative controls, respectively. Finally, DNA sequences were determined using the dideoxy-chain-termination method with a commercial DNA sequencing kit (BigDye™ Terminator v3.1 Cycle Sequencing Kit, Applied Biosystems™ (Thermo Fisher Scientific, Waltham, MA, USA) according to the manufacturer’s instructions. The nucleotide sequences obtained were identified using the Basic Local Alignment Search Tool (BLAST).

### 2.2. Determination of Minimum Inhibitory Concentration (MIC)

For each *E. coli* isolate, we tested their antibiotic susceptibility using the Minimum Inhibitory Concentration (MIC) method [22]. MIC values (µg/mL) were determined using commercial plates (96-well Sensititre™Plate Thermo Scientific, Waltham, MA, USA) containing scaled dilutions (2-fold dilutions) of eight antimicrobials used in Italy in both veterinary and human medicine [23]. The antibiotics and their dilutions were: ampicillin, 0.25–32 µg/mL (AMP)*;* amoxicillin/clavulanic acid 2:1 ratio, 0.25/0.12–32/16 µg/mL (AUG2)*;* cefazolin, 0.5–8 µg/mL (FAZ)*;* cefotaxime, 0.5–4 µg/mL (FOT); tetracycline, 1–16 µg/mL (TET)*;* colistin, 0.03–8 µg/mL (COL)*;* gentamicin, 0.25–32 µg/mL (GEN)*;* trimethoprim/sulfamethoxazole, 0.06/1.19–8/152 µg/mL (SXT) (Thermo Scientific, Waltham, MA, USA). In accordance with the manufacturer’s instructions, 10 µL of 0.5 McFarland turbidity bacterial suspension were mixed into 10 mL of Mueller–Hinton broth (Thermo Scientific, Waltham, MA, USA). After dispensing 50 µL of inoculum into each well, the plate was incubated at 37 °C for 18–24 h. The *E. coli* ATCC 25922 strain was used as a quality control for the MICs. After the incubation time, a manual reading was performed using Sensititre ™ Manual Viewbox (Thermo Scientific, Waltham, MA, USA) and the results were interpreted in accordance with Clinical and Laboratory Standards Institute (CLSI) breakpoints [24,25].

### 2.3. Detection of Antibiotic Resistance Genes

Based on the MIC results, we tested each antibiotic-resistant isolate for the presence of β-lactamases and tetracycline resistance genes using previously published methods [26,27]. End-point PCR protocols were adapted to Real-Time PCR to optimize cost and reduce analysis time to detect *bla*_TEM_, *bla*_CTX-M_, *tet*(A) and *tet*(B) genes. The analyses were performed using the CFX96 Touch Real-Time PCR Detection System (Bio-Rad Laboratories, Hercules, CA, USA). The reaction mixture was prepared using 10 ng of sample DNA, 0.5 µM of forward and reverse primers listed in Table 1, in a total volume of 25 µL of Advanced Universal SYBR Green Supermix 1X (Bio-Rad Laboratories, Hercules, CA, USA).

The amplification program included an initial denaturation at 94 °C for 10 min, followed by 32 cycles of 94 °C for 30 s, 60 °C for 30 s, and 72 °C for 15 s, and final extension at 72 °C for 10 min. DNA from a previously sequenced *E. coli* harboring *bla* and *tet* gene was used as a positive control. Negative control was a No Template Control (NTC), in which the template DNA was replaced with DNAse free water to obtain the final volume. Finally, 10 µL of the PCR product was used for electrophoresis on E-Gel™ Go! Agarose Gels, 2% (Thermo Fisher Scientific, Waltham, MA, USA) to confirm the size of the product.

## 3. Results

PCR conducted on the 75 *E. coli* isolated from cats revealed the presence of resistance genes in 43% of the strains. Notably, 29% of the strains were found to harbor the *bla*_TEM_ gene, 23% the *bla*_CTXM_, 21% the *tet*(A), and 20% the *tet*(B).

For the evaluation of phenotypic susceptibility patterns, the MIC assay was conducted, which also showed that 43% of the *E. coli* isolates were resistant to at least one of the eight antibiotics tested (Table 2). Specifically, 31% of the isolates were resistant to ampicillin, 23% to cefotaxime, 21% to tetracycline, 20% to cefazolin and 17% to amoxicillin/clavulanic acid. Furthermore, six (19%) of the 32 resistant isolates were multidrug resistant (MDR), being resistant to at least one agent from three classes of antibiotics [28], namely penicillins, cephalosporins and tetracyclines. Among isolates resistant to at least one antibiotic, isolates were resistant to a median of 2.5 antibiotics [mean 2.2, range: 1–5]. Likewise, antibiotic resistant isolates harbored a median of 2.5 resistance genes [mean 2.5, range: 1–4]. In contrast, no isolates showed resistance to colistin, gentamicin and trimethoprim/sulfamethoxazole.

## 4. Discussion

The role of animals as carriers and potential reservoirs of AMR is a growing concern worldwide [29]. Several studies evaluating the circulation of AMR among animals have focused on farm animals, companion animals, and wildlife, but little information is available on stray cats and dogs [30]. In this study, fecal carriage of antibiotic-resistant *E. coli* was detected in 43% of stray cats sampled during a sterilization program carried out in Sicily during 2022. The highest phenotypic antibiotic resistance prevalence was observed for ampicillin, while all antibiotic-resistant isolates harbored at least one gene coding for resistance to beta-lactams or tetracycline antibiotics, both critically important to public health. Stray cats are synanthropic animals that encounter and share their urban environment with humans. This study highlights the need to better understand their potential role as AMR reservoirs for humans and their own pets. The law in Italy provides that these animals are captured and placed in catteries, where they receive all the necessary care while waiting to be adopted, or managed by animal welfare associations or volunteers in feline colonies, i.e., groups of cats that live freely and usually stay in the same place [21]. These animals, which are on the borderline between domestic and stray animals, could therefore play an important role in the circulation of zoonotic diseases and antimicrobial resistance, especially in the spread of pathogens over distances greater than those traveled by owned cats [31].

Tetracycline and beta-lactam antibiotics are widely used to treat bacterial infections in companion animals [32]. Therefor, resistance to these antibiotics can reduce the efficacy of the treatment of several bacterial infections affecting cats. In agreement with previous studies conducted on dogs and cats in Italy, our study showed that the *bla*_TEM_ gene was the most detected antibiotic resistance gene among *E. coli* isolates, followed by *tet*(A) and *bla*_CTX-M_ [29,30,33,34]. In addition, all strains that harbouring at least one *bla* or *tet* gene were also resistant to at least one antibiotic of the corresponding class (Table 2). Similar to previous studies, isolates harbouring the *bla*_TEM_ and *bla*_CTX-M_ genes alone or in combination were resistant to ampicillin, first- and third-generation cephalosporins, and amoxicillin/clavulanic acid, which could indicate the presence of ESBL in the studied cats [35,36,37]. Although we did not sequenced the genotypes identified and the TEM group can also include narrow-spectrum beta-lactamases, the genotype CTX-M only refers to extended-spectrum beta-lactamases (ESBL) [38].

Our findings reinforce the importance of implementing One Health national AMR monitoring plans for companion animals, that include the surveillance of phenotypic and genotypic resistant profiles for critically important antibiotics in both human and animal health [39]. It is well known that, unlike most diseases, antimicrobial resistance can be transmitted from host to host, even with low numbers of bacteria, and that exchanges of antibiotic-resistance genes (ARGs) can occur between bacteria from animals and humans, as well as through the environment [39]. Thus, a more careful monitoring of AMR in owned and stray companion animals living on the border between urban and wild environments is necessary to understand the routes of dissemination of ARGs, and plan effective strategies to reduce the burden of AMR.

Despite our main conclusions, we acknowledge several limitations in this study that could be complemented by future research. For example, we lacked information on the history of previous antimicrobial treatments in these animals, which cannot be excluded considering that some stray cats may have been owned and then abandoned. As a result, it is unclear whether resistance to some types of antimicrobials can be attributed to misuse or whether ARGs result by acquisition from an unknown human or environmental source. Since we focused on screening a limited number of common resistance genes, we also cannot exclude the involvement of other resistance mechanisms for the phenotypic resistance to beta-lactams, such as efflux pumps, pore deficiencies, expression of different ESBL genes, and mutations in the chromosomal AmpC gene [40]. Finally, future research on the molecular typing of *E. coli* isolates could provide further insights on which clones circulating, whether ARGs are carried by mobile genetic elements such as plasmids, and whether these *E. coli* isolates carry virulent factors associated with potentially pathogenic strains.

## 5. Conclusions

This study showed that stray cats can be fecal carriers of antibiotic resistant *E. coli*. Stray cats could spread antibiotic-resistant bacteria and ARGs in the urban environment or, if being adopted, within households. Thus, the risk of cross-transmission of antibiotic-resistant bacteria as well as the emergence and spread of AMR in these animals should be considered a challenge for veterinary medicine, both from an animal and public health perspective. This study highlights the need for a systematic One Health surveillance including these animals to better understand the mechanisms of transmission of antimicrobial resistance across animals, humans, and the environment.

## Figures and Tables

**Table 1:** Primer used in this study.

Target	Primers	Sequence (5′–3′)	Amplicon Size (bp)	References
*bla*_TEM_	*bla*_TEM__F	TTCCTGTTTTTGCTCACCCAG	112	[26]
	*bla*_TEM__R	CTCAAGGATCTTACCGCTGTTG		
*bla*_CTX-M_	*bla*_CTX-M__F	CTATGGCACCACCAACGATA	103	
	*bla*_CTX-M__R	ACGGCTTTCTGCCTTAGGTT		
*tet*(A)	*tet*(A)_F	GCTACATCCTGCTTGCCTTC	210	[27]
	*tet*(A)_R	CATAGATCGCCGTGAAGAGG		
*tet*(B)	*tet*(B)_F	TTGGTTAGGGGCAAGTTTTG	659	
	*tet*(B)_R	GTAATGGGCCAATAACACCG		

**Table 2:** Antibiotic resistance patterns of *E. coli* from cats.

Phenotypic Patterns	Genetic Patterns	Number of Strain
AMP-AUG2	*bla*_TEM_	7
FAZ-FOT	*bla*_CTXM_	5
AMP-TET	*bla_TEM_-tet(A)-tet(B)*	5
AMP- AUG2-FAZ-FOT-TET	*bla*_TEM_-*bla*_CTXM_*-tet*(A)-*tet*(B)	4
AMP-FAZ-FOT	*bla*_TEM_-*bla*_CTXM_	4
TET	*tet*(A)-*tet*(B)	3
AMP-AUG2-FAZ-FOT-TET	*bla*_TEM_-*bla*_CTXM_-*tet*(A)	2
FOT-TET	*bla*_CTXM_*-tet*(A)-*tet*(B)	2

Ampicillin, (AMP); amoxicillin/clavulanic acid 2:1 Ratio, (AUG2); cefazolin, (FAZ); cefotaxime, (FOT); tetracycline, (TET).

## Data Availability

All data discussed are contained in the manuscript.
